# The Immunomodulatory Effect of Acupoint Application for Childhood Asthma: A Systematic Review and Meta-Analysis

**DOI:** 10.1155/2015/896247

**Published:** 2015-04-27

**Authors:** Xiao Cun Yang, Tao Yin, Qian Gao, Ling Jun Kong

**Affiliations:** ^1^Yueyang Hospital of Integrated Traditional Chinese and Western Medicine, Shanghai University of Traditional Chinese Medicine, Shanghai 201203, China; ^2^Department of Rehabilitation, The Central Hospital of Tai'an, Tai'an, Shandong 271000, China; ^3^Department of Tuina, The Second Hospital of Traditional Chinese Medicine in Jiangsu Province, Nanjing, Jiangsu 210017, China

## Abstract

*Objective*. To evaluate the evidence on the immunomodulatory effect of acupoint application for childhood asthma. *Methods*. Five electronic databases through October 2014 were searched. The risk of bias in eligible studies was assessed using the Cochrane Collaboration tool. Standardised mean difference (SMD) and 95% confidence intervals (CI) of random-effects model were calculated. And heterogeneity was assessed using the Cochran *Q* statistic and quantified with the *I* ^2^ index. *Results*. Six studies were included in our review. The aggregated results suggested that acupoint application showed the beneficial effect for childhood asthma in improving IgA (SMD, −0.83; 95% CI −1.14 to −0.52; *P* < 0.00001), IgE (SMD, −0.52; 95% CI −0.76 to −0.29; *P* < 0.001), IgG (SMD, −1.17; 95% CI −1.61 to −0.74; *P* < 0.0001), IL-4 (SMD, −0.57; 95% CI −0.91 to −0.23; *P* = 0.0009), and IFN-*γ* (SMD, −0.38; 95% CI −0.71 to −0.04; *P* = 0.03) but not IgM (SMD, −0.40; 95% CI −0.98 to 0.18; *P* = 0.18). And the effective dose of acupoint application may be 2–6 hours/time and a total of 3 times within 4 weeks. *Conclusions*. This review showed the positive evidence that acupoint application had the favorable immunomodulatory effect for childhood asthma. However, more studies with long follow-up are warrant to confirm the current findings.

## 1. Introduction

Asthma is one of the most common chronic respiratory diseases among children, which imposes a substantial care burden on families and health services [1–3]. In United States, nearly 6 million children suffer from asthma [3]. In recent years, with rapidly deteriorating air quality, the incidence of childhood asthma is between 3.47% and 10.6% in China [4–8].

Common starting medications for childhood asthma are effective such as short-acting beta2-receptor agonists and inhaled corticosteroids, but adverse drug reactions are still considerable [9]. Inhaled corticosteroids can result in slower growth of asthmatic children [10–12]. Therefore, complementary and alternative treatments with fewer adverse effects are employed by more children with asthma.

Acupoint application, as a complementary and alternative treatment for asthma, is a kind of herbal cone cake fixed into acupoints (Tiantu “CV 22,” Fei Shu “BL 13,” Dazhui “CV 14,” etc.). The herbal cone cake is mixed with herbal powders (Rou Gui “*Cortex Cinnamomi,*” Ding Xiang “*Flos Caryophylli,*” Wu Zhu Yu “*Fructus Evodiae,*” etc.) with ginger juice [13]. It showed favorable effects in prolonging clinical remission of asthma [14, 15]. In the light of these benefits, the researchers have sought to better understand the role of acupoint application on the immune system. Some studies [16–18] reported the positive immunomodulatory effect of acupoint application for childhood asthma, but it remained less defined.

Therefore, the purpose of this review is to evaluate the evidence on the immunomodulatory effect of acupoint application for childhood asthma. To our knowledge, this is the first comprehensive review to summarize the efficacy of acupoint application on the immune system focusing on immunoglobulin, interleukin, interferon, and so forth. Based on our findings, recommendations for future research are offered.

## 2. Materials and Methods

### 2.1. Search Strategy

The following electronic databases through October 2014 were searched: PubMed, EMBASE, Cochrane Library, China Knowledge Resource Integrated Database, and Wan Fang Data. The following keywords were used: acupoint application and asthma. The reference lists of selected reviews were also screened. In order to identify unpublished studies, dissertations and trial registrations were searched. And we contacted experts in the field.

### 2.2. Study Selection

The studies that met the following criteria were included: (1) study design was randomized controlled trials (RCTs); (2) the target population was children diagnosed with asthma; (3) the main intervention should be acupoint application compared with any comparator without acupoint application; (4) the immune outcomes included immunoglobulin, interleukin, and interferon; (5) the study was available in either English or Chinese.

Identified abstracts were screened independently by two authors. All full-texts of potentially relevant abstracts were evaluated based on eligibility criteria by one author and confirmed by the other author.

### 2.3. Data Extraction

Data extraction was performed by two authors independently. They extracted information on the characteristics of study and population, immune outcomes, the follow-up period, and dose of interventions. The corresponding author was contacted when relevant information was not reported. And disagreements were resolved by consensus among authors.

### 2.4. Risk of Bias Assessment

The risk of bias in eligible studies was assessed independently by two authors using the Cochrane Collaboration tool [19, 20]. Six domains were evaluated: (1) random sequence generation, (2) allocation concealment, (3) blinding of outcome assessment, (4) incomplete outcome data, (5) selective reporting, and (6) other bias. The authors paid attention to blinding of outcome assessment in assessing blinding methods because it was difficult to blind participants and therapists for therapeutic methods in alternative therapy studies. Every domain could be classified as “low risk of bias,” “high risk of bias,” or “unclear risk of bias.” And the corresponding author was contacted when relevant information was not reported. There was no disagreement among authors regarding bias assessment of eligible studies.

### 2.5. Data Synthesis and Analysis

For continuous data, standardized mean difference (SMD) and 95% confidence intervals (CI) of random-effects model were calculated for all eligible trials. Heterogeneity was assessed with the Cochran *Q* statistic (considered significant when *P* value was less than 0.10) and quantified with the *I* ^2^ index (considered high when *I* ^2^ was above 75%). Trials, including 2 similar control groups, had the groups combined with the formula provided by the Cochrane handbook for a single pairwise comparison. All meta-analyses were conducted using Cochrane Collaboration software (Review Manager Version 5.2).

## 3. Results

### 3.1. Literature Search

**Figure 1:**
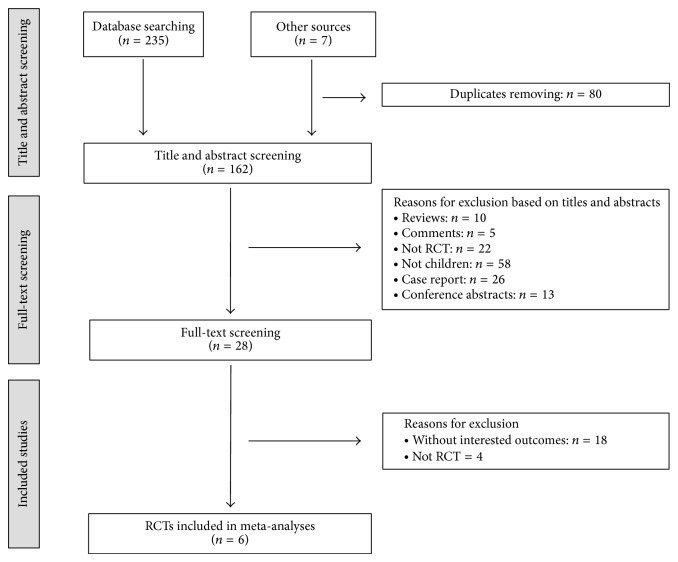
Flow diagram of study selection. RCTs: randomized controlled trials.

The process of literature search and study selection was depicted in Figure 1. After removing duplicates, 162 records were identified. 28 full-texts were retrieved in screening full-texts. At last, 6 RCTs [16–18, 21–23] were included in our reviews. 22 full-texts were eliminated due to being without interested outcomes (*n* = 18) and not RCTs (*n* = 4).

### 3.2. Study Characteristics

6 RCTs [16–18, 21–23] of acupoint application for childhood asthma were included in our review. All eligible studies were conducted in China between 2003 and 2012. A total of 564 participants with mean age of 6.79 years were included in eligible studies. The duration of acupoint application was 4 weeks in all eligible studies. The sessions of acupoint application ranged from 3 to 10. And the time of each acupoint application ranged from 2 to 26 hours. The characteristics of all eligible studies were summarized in Table 1.

### 3.3. Risk of Bias

**Figure 2:**
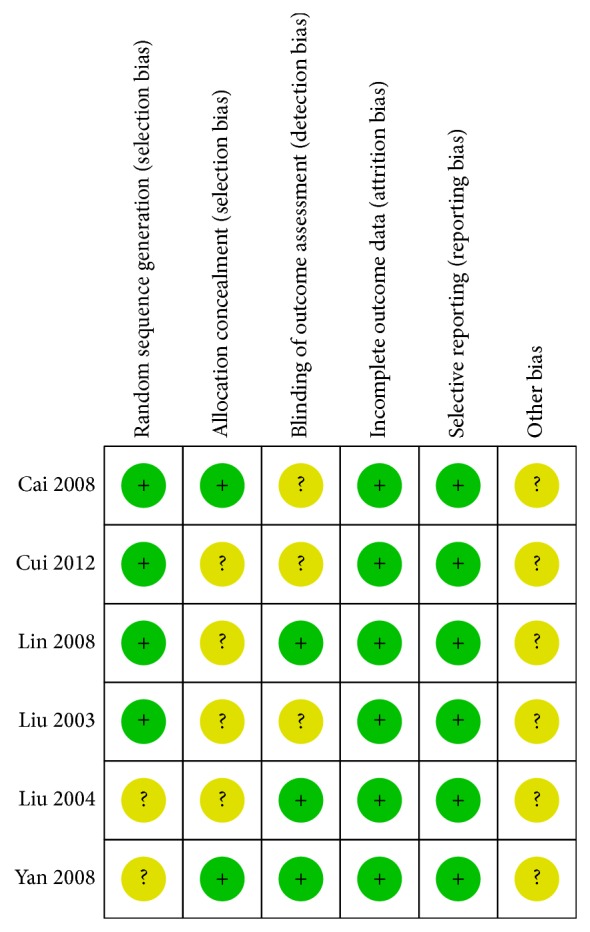
Risk of bias. Yellow (?): unclear risk of bias; green (+): low risk of bias.

The risk of bias evaluation is reported in Figure 2. Four trials [16, 18, 22, 23] employed appropriate random sequence generation including random number table and computer generation. Four studies [16–18, 23] did not report detailed descriptions on whether allocation concealment was performed. The independent assessor was employed in three trials [17, 18, 21], while it was unclear in other ones [16, 22, 23]. And all studies [16–18, 21–23] were categorized as low risk of bias in incomplete outcome data and selective reporting.

### 3.4. Quantitative Data Synthesis

**Figure 3:**
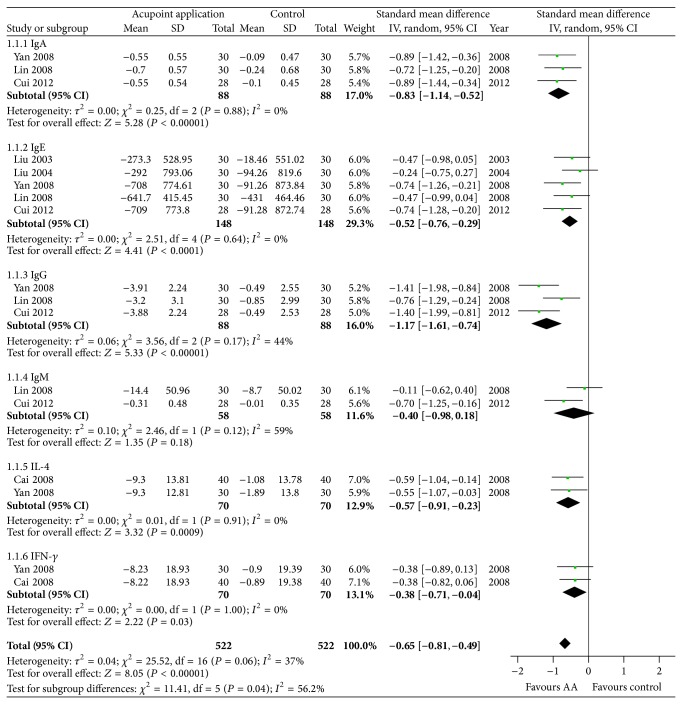
Forest plot showing the effect of acupoint application for childhood asthma on the immune system. IgA: immunoglobulin A, IgE: immunoglobulin E, IgG: immunoglobulin G, IgM: immunoglobulin M, IL-4: interleukin-4, IFN-*γ*: interferon-*γ*, and AA: acupoint application.

Six studies [16–18, 21–23] reported the efficacy of acupoint application for childhood asthma on immune outcomes including immunoglobulin A (IgA), immunoglobulin E (IgE), immunoglobulin G (IgG), immunoglobulin M (IgM), interleukin-4 (IL-4), and interferon-*γ* (IFN-*γ*). The results of our meta-analysis suggested that acupoint application showed significant improvements on immune outcomes (SMD, −0.65; 95% CI −0.81 to −0.49; *P* < 0.00001, Figure 3).

### 3.5. IgA

Three studies [18, 21, 23] evaluated the effect of 4-week acupoint application for childhood asthma on IgA. All of them reported beneficial effects of acupoint application on IgA and were included in our meta-analysis. The aggregated result also suggested that acupoint application showed the significant improvement on IgA compared with control interventions (SMD, −0.83; 95% CI −1.14 to −0.52; *P* < 0.00001, Figure 3) [18, 21, 23].

### 3.6. IgE

Five studies [16–18, 21, 23] evaluated the effects of 4-week acupoint application for childhood asthma on IgE. All of them (444 participants) were included in our meta-analysis. Although three studies [16–18] did not report significant difference between acupoint application and control interventions on IgE, the aggregated result of the meta-analysis supported the idea that acupoint application showed statistically significant improvements on IgE (SMD, −0.52; 95% CI −0.76 to −0.29; *P* < 0.0001, Figure 3) [16–18, 21, 23].

### 3.7. IgG

For IgG, three studies [18, 21, 23] with a total of 264 participants were included in the meta-analysis. And our meta-analysis showed that 4-week acupoint application demonstrated better effect on IgG compared with control interventions (SMD, −1.17; 95% CI −1.61 to −0.74; *P* < 0.00001, Figure 3) [18, 21, 23].

### 3.8. IgM

Two studies [18, 24] assessed the effects of 4-week acupoint application on IgM. All of them (144 participants) were included in our meta-analysis. And the aggregated result demonstrated a small but not statistically significant effect on IgM (SMD, −0.40; 95% CI −0.98 to 0.18; *P* = 0.18, Figure 3) [18, 24].

### 3.9. IL-4

Two trials [21, 22] were included in our meta-analysis of acupoint application for childhood asthma on IL-4. Both of them reported that acupoint application showed better effects on IL-4 than control interventions. And the meta-analysis also supported favorable effects of 4-week acupoint application on IL-4 (SMD, −0.57; 95% CI −0.91 to −0.23; *P* = 0.0009, Figure 3) [21, 22].

### 3.10. IFN-*γ*

Two studies [21, 22] evaluated the effects of 4-week acupoint application on IFN-*γ*. Both of them with 240 participants were included in our meta-analysis. Although two studies did not report significant difference between acupoint application and control interventions, the aggregated result showed that 4-week acupoint application demonstrated medium effect and statistically significant improvements on IFN-*γ* (SMD, −0.38; 95% CI −0.71 to −0.04; *P* = 0.03, Figure 3) [21, 22].

### 3.11. Follow-Up Effects

Only one study reported that asthmatic reoccurrence of children with acupoint application became less frequent in 24-week follow-up [16].

### 3.12. Adverse Events

No serious adverse events were reported in included studies of acupoint application for childhood asthma.

## 4. Discussion

This review extends the previous investigations [14, 15] of the efficacy of acupoint application on health outcomes, focusing on the promising role that it may play in regulating the immune system. Overall, our findings suggested that acupoint application showed positive effects in regulating IgA, IgE, IgG, IL-4, and IFN-*γ* for asthmatic children. Although there was not sufficient evidence to support or refute the value of acupoint application in regulating IgM, current findings may provide insight into the potential mechanisms behind acupoint application and the health benefits it confers.

Acupoint application is widely accepted as a preventive treatment for childhood asthma in China, but to our knowledge, this is the first comprehensive review to evaluate the immunomodulatory effects of acupoint application for childhood asthma. Based on included studies, acupoint application significantly improved immune outcomes including IgA, IgE, IgG, IL-4, and IFN-*γ* in asthmatic children. Previous systematic reviews have assessed the effect of acupoint application on symptom scores of childhood asthma and reported that it showed favorable effects in prolonging clinical remission of asthma [14, 15]. Although some studies reported that acupoint application showed beneficial improvements on lung function for adults with asthma [25, 26], few studies assessed the effect of acupoint application on lung function of childhood asthma. The reason may be that it is difficult to test the lung function for children.

The therapeutic dose of acupoint application is important for asthma. In 83% eligible studies, 4-week acupoint application was employed. And there were 2–6 hours each time and a total of 3 times during 4 weeks. Therefore, the acupoint application (2–6 hours, 3 times within 4 weeks) should be recommended as a complementary and alternative treatment for childhood asthma, especially in improving immunomodulatory function. And the acupoints usually contain Tiantu “CV 22,” Fei Shu “BL 13,” Dazhui “CV 14,” Gaohuangshu “BL 43,” and so forth.

Assuming that acupoint application was effective for asthma, some complex immunomodulatory mechanisms may provide possible rationales. IgE was considered a potent predictor of the development of asthma and played a central role in the pathophysiology of asthma [27]. Several studies reported that total serum IgE level was higher in asthmatic children [24, 28, 29]. And relevant meta-analysis showed that IL-4 was significantly associated with asthma [30]. Some studies also supported the importance of IL-4 for childhood asthma [31–33]. What is more, there was a significant correlation between IL-4 and IgE in asthmatic subjects [34, 35]. It has been shown that IL-4 played a critical role in IgE synthesis [36]. In addition, IFN-*γ* was thought to protect against the development of asthma by regulating T helper-2 cytokine production [37]. And the serum IL-4 level may be elevated in concern with decreased IFN-*γ* level in patients with asthma [38]. Therefore, the regulating effect on IgA, IgE, IgG, IL-4, and IFN-*γ* may be the basic mechanism of acupoint application for childhood asthma.

Some relevant studies also supported our findings. In the review of herbal interventions for asthma in adults and children, the authors concluded that herbal preparations showed the valuable efficacy for asthma [39, 40]. And some studies also reported that Chinese herbs exhibited specific anti-inflammatory and immunoregulatory effects in a chronic asthmatic mice model [41–43]. Acupoint application, as a complementary and alternative treatment for asthma, is a kind of herbal cone cakes which are applied to acupoints. So the studies of herbal interventions for asthma provided indirect evidence for acupoint application in the management of asthma.

### 4.1. Limitations

There were several limitations in our review. First, all eligible studies were conducted in China, which limit the generalizability of our findings due to population characteristics. Second, there were only two eligible trials in some subgroup meta-analyses due to strict eligibility criteria. It may bias aggregated results, but low eligibility criteria would generate more doubtful results. Third, the eligible studies did not focus on lung function of children with asthma because it is difficult to test lung function of children. However, it may affect physicians' decisions of acupoint application for childhood asthma because the improvement of lung function is important for childhood asthma. In addition, serious adverse events were not reported in eligible trials, but it should be careful to conclude that acupoint application was safe because it was not clear whether adverse effects had been measured or not in some studies. These limitations, especially in population characteristics and fewer eligible studies, may affect our findings and restrict the applied range of acupoint application.

## 5. Conclusions

Our review showed the positive evidence that acupoint application had favorable immunomodulatory effects for childhood asthma, especially on IgA, IgE, IgG, IL-4, and IFN-*γ*. And the effective dose of acupoint application may be 2–6 hours/time and a total of 3 times within 4 weeks. Considering few included studies in some subgroup meta-analyses and lack of follow-up data, more RCTs with long follow-up are warrant to confirm the current findings.

## Figures and Tables

**Table 1:** Characteristics of the included studies.

First author, year	Sample size	Mean age (years)	Follow-up (weeks)	Main outcomes	Experimental group intervention	Control group intervention
Liu 2003 [16]	120	6.5	24	IgE	Acupoint application (2–4 h/3 sessions/4 weeks)	(1) Usual care (2) Ketotifen (0.5–1 mg, Bid, 8 weeks)

Liu 2004 [17]	60	8.21 ± 2.58 8.15 ± 2.74	—	IgE	Acupoint application (26 h/3 sessions/4 weeks)	Usual care

Lin 2008 [18]	60	5.68 ± 2.34	—	IgA, IgE, IgG, and IgM	Acupoint application (2–6 h/10 sessions/4 weeks)	Pulmicort (200–400 *μ*g, Qd, 4 weeks) plus ketotifen (0.5–1 mg, Bid, 4 weeks)

Yan 2008 [21]	120	6.75 ± 0.77 6.76 ± 0.76 6.72 ± 0.79 6.78 ± 0.75	—	IgA, IgE, IgG, IL-4, and IFN-*γ*	Acupoint application (2–6 h/3 sessions/4 weeks)	(1) Usual care (2) Pulmicort (200–600 *μ*g, Qd, 12 weeks) (3) Ketotifen (0.5–1 mg, Bid, 12 weeks)

Cai 2008 [22]	120	6.76 ± 2.76 6.77 ± 2.75 6.77 ± 2.73	—	IL-4 and IFN-*γ*	Acupoint application (24 h/3 sessions/4 weeks)	(1) Usual care (2) BCG-PNA (1-2 mg/24 sessions, 12 weeks)

Cui 2012 [23]	84	6.22 ± 2.56 6.72 ± 2.76 6.33 ± 2.72	—	IgA, IgE, IgG, and IgM	Acupoint application (4–6 h/3 sessions/4 weeks)	(1) Usual care (2) Pulmicort (200–600 *μ*g, Qd, 48 weeks)

IgE: immunoglobulin E; IgA: immunoglobulin A; IgG: immunoglobulin G; IgM: immunoglobulin M; IL-4: interleukin-4; IFN-*γ*: interferon-*γ*; BCG-PNA: BCG polysaccharide and nucleic acid injection.
